# Treatment of patients with geriatric depression with repetitive transcranial magnetic stimulation

**DOI:** 10.1007/s00702-019-02037-5

**Published:** 2019-06-27

**Authors:** F. Leblhuber, K. Steiner, Dietmar Fuchs

**Affiliations:** 1 Department of Gerontology, Kepler University Clinic, Linz, Austria; 2 Division of Biological Chemistry, Biocenter, Innsbruck Medical University, Innrain 80, 4th Floor, Room M04-313, 6020 Innsbruck, Austria

**Keywords:** Geriatric depression, Transcranial magnetic stimulation, Phenylalanine hydroxylase, Tryptophan metabolism, Neopterin

## Abstract

Repetitive transcranial magnetic stimulation (rTMS) has become a useful tool to treat different neuropsychiatric conditions such as depression, dementia and extrapyramidal syndromes insufficiently responding to conventional treatment. In this SHAM-controlled exploratory study safety, symptom improvement as well as changes in inflammation markers and neurotransmitter precursor amino acids availability were studied after a prefrontal cortex (PFC) stimulation using rTMS as add-on treatment in 29 patients with geriatric depression. Out of these, ten patients received SHAM treatment. Treatment was well tolerated, no serious adverse effects were observed. A clear improvement in symptoms of depression with a significant decrease in the HAMD-7 (*U* = 3.306, *p* = 0.001) was found by rTMS treatment. In parallel, serum phenylalanine dropped significantly (*U* = 2.340, *p* < 0.02), and there was a decline of tryptophan and of Phe/Tyr concentrations, both the effects, however, failed to reach the levels of statistical significance. In the patients who underwent SHAM treatment, no significant changes of HAMD-7 or the concentrations of any biomarker in the study could be found. In addition to the significant effect of rTMS on depression scores, these results point to a possible influence of rTMS on the enzyme phenylalanine hydroxylase (PAH), which plays a crucial role in the biosynthesis of neurotransmitter precursors related to geriatric depression.

**Electronic supplementary material:**

The online version of this article (10.1007/s00702-019-02037-5) contains supplementary material, which is available to authorized users.

## Introduction

Repetitive transcranial magnetic stimulation (rTMS) is a promising treatment for depressive disorders, delivering focused magnetic field pulses to the brain, it can influence brain function if delivered repetitively. The magnetic field induced by rTMS can excite or inhibit a small brain area, thus altering cortical excitability (Hallet 2007; Noda et al. 2015). Previous studies described rTMS as effective intervention in depressive illness, especially as augmentation in treatment-resistant cases (Liu et al. 2014, 2017).

Currently electroconvulsive therapy (ECT) is considered as the most effective treatment for depressive disorders, but this method involves the administration of anaesthetics and muscle relaxants to avoid convulsions. rTMS does not involve a seizure, and this procedure is associated with minimal side-effects (Koren et al. 2001; Bakker et al. 2015). rTMS over the prefrontal cortex (PFC) regulates the processing of emotion and mood (Liu et al. 2017), but wide-ranging brain regions play distinct roles in the pathophysiology of this affective disorder (Dalgleish 2004; Pessoa 2017). Earlier data indicate lateralisation of emotional processing, whereby the right hemisphere predominantly processing negative and the left hemisphere processing positive affects (Prete et al. 2015). Furthermore, new potential targets for rTMS in controlling emotional processing in depression are described (Downar and Daskalakis 2013) such as the dorsomedial prefrontal cortex (DMPFC), frontopolar cortex (FPC), ventromedial prefrontal cortex (VMPFC) and ventrolateral prefrontal cortex (VLPFC).

One of the biological risk factors of late-life depression is immune activation (Dantzer et al. 2002; Widner et al. 2002; Tiermeier 2013; Capuron et al. 2011). Chronic low-grade inflammation in ageing is associated with alterations of tryptophan and tyrosine metabolism (Capuron et al. 2011). On the one hand, the pro-inflammatory cytokine interferon-γ stimulates the biosynthesis of tetrahydrobiopterin (BH4), which is rate-limiting for the biosynthesis of the neurotransmitters serotonin, dopamine, adrenaline and noradrenaline (Neurauter et al. 2008). On the other hand, interferon-γ triggers the high output of reactive oxygen species in macrophages, which destroys the oxidation-labile BH4. Recent data suggest that oxidative loss of BH4 triggered by interferon-γ can reduce the biosynthesis of catecholamines, which may relate to disturbed neurotransmitter pathways in depression (Neurauter et al. 2008; Sperner-Unterweger et al. 2014). In this exploratory intervention study, the effect of rTMS on depression scores and on serum concentrations of immune system biomarker neopterin and neurotransmitter precursor amino acids correlated to late-life depression was investigated.

## Patients and methods

From 55 consecutive outpatients from the Department of Gerontology of the Neuromed Campus at the Kepler University Clinic Upper Austria with different neuropsychiatric symptomatology (somatoform, depressive and anxiety disorders, addiction, paranoia and delirious syndromes, progressive cognitive decline and dementia), 29 patients (aged mean ± SEM: 72.4 ± 2.10 years, 16 females) symptoms of treatment-resistant depression (incomplete remission of depressive symptoms after adequate antidepressant treatment, Thase 2011) were included in this study. On weekdays, they underwent 10 subsequent active (*n* = 19: 71.9 ± 2.92 years) or SHAM (*n* = 10: 73.3 ± 2.69 years) rTMS stimulations of the PFC bilaterally with a magnetic loop of a Theracell^®^ apparatus (Guth Meditec, Salach, Germany; frequency 3 Hz, 0.08 T, duration of treatment 30 min) in a randomised order at a ratio of 2:1. Stimulation of the PFC was adjusting the intensity above the individual motor threshold to elicit visible bilateral contractions of the mimic musculature in the *verum* group (Vamava et al. 2011). SHAM treatment was performed setting the magnetic loop of the Theracell apparatus to 3 Hz, 0.00 Tesla, duration of treatment was 30 min.

All patients were investigated as described earlier including routine laboratory tests and cerebral magnetic resonance tomography (MRT) to exclude circumscript cerebral lesions (Leblhuber et al. 2018). Clinical assessment was performed before and after intervention, using the 7-item Hamilton depression scale (HAMD-7; McIntyre et al. 2005). Medication including antidepressants was given constantly throughout rTMS treatment. Patients included did not receive any psychotherapy during this intervention.

The following parameters were controlled before and after rTMS treatment: serum concentrations of neopterin, tryptophan and kynurenine, calculating the kynurenine to tryptophan ratio (Kyn/Trp) as an index of tryptophan breakdown (Widner et al. 1997; Leblhuber et al. 2018), and tyrosine and phenylalanine, calculating the phenylalanine to tyrosine ratio (Phe/Tyr) as an index of phenylalanine hydroxylase (PAH) activity (Neurauter et al. 2013). Measurements were performed immediately before first/after last rTMS treatment and thus not in a fasted state. Waist circumference was within 90 and 105 cm (males and females), there were neither clinical nor laboratory signs of malnutrition.

Data were analysed by the Statistical Package for Social Science (version 19, SPSS, Chicago, IL, USA) as described earlier (Leblhuber et al. 2018). To take into account that not all collected data followed a normal distribution, non-parametric Friedman and Wilcoxon signed-rank test were applied. To test for associations between variables, Spearman rank correlation analysis was performed. ANOVA with repeated measurements was applied, as between-grouping factors “group” VERUM (patients) vs. SHAM (controls) were chosen, as within-grouping factor “phase” time points before and after treatment. In each ANOVA, number of observation was 58. For the overall models, there were 30 degrees of freedom, which were divided into 1 degree of freedom each for “treatment”, “time” and the interaction term “time#treatment”; the remaining 27 degrees of freedom being associated with the residual error. Calculations were performed with statistical package “STATA”, version 14.2 (StataCorp LLC, 4905 Lakeway Drive, College Station, Texas 77845, USA). *p* values below 0.05 were considered to indicate significance.

The study was approved by the local ethics committee. Patients were treated with rTMS after informed consent according to the declaration of Helsinki.

## Results

**Fig. 1:**
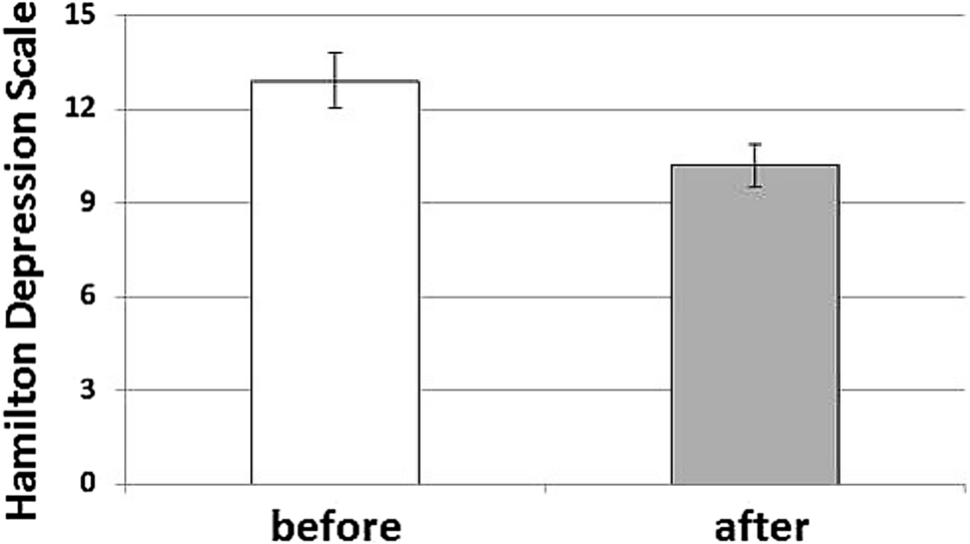
HAMD-7 depression scale in 19 patients with geriatric depression before and after 10 sessions of rTMS treatment (mean values ± SEM are shown; *U* = 3.306; *p* < 0.001)

Routine laboratory tests including leucocyte count and C-reactive protein showed results within normal limits. rTMS intensity is well-tolerated without any serious adverse events (Vamava et al. 2011). rTMS induced significant depression symptom improvement and a significant decrease in the HAMD-7 score after active treatment (mean ± SEM, before 12.9 ± 0.89, after: 10.2 ± 0.67; *U* = 3.306, *p* = 0.001, Fig. 1). No effect was found in the SHAM-treated group (13.2 ± 1.43 before, after: 13.3 ± 1.48; *U* = 0.447, n.s.).

In the rTMS-treated group (*n* = 19), average concentration of tryptophan before intervention was 58.6 ± 3.56 nmol/L, of kynurenine 1.85 ± 0.10 μmol/L, of Kyn/Trp, 33.3 ± 2.53 μmol/mmol, of tyrosine 83.9 ± 5.82 μmol/L, of phenylalanine 98.2 ± 7.22 μmol/L, and of Phe/Tyr was 1.19 ± 0.051 µmol/µmol. The average neopterin concentration was 10.6 ± 0.79 nmol/L and nitrite concentration was 45.8 ± 7.65 μmol/L (see Table 1).

**Table 1:** Serum concentrations (mean values ± SEM) of neopterin, nitrite and neurotransmitter precursor amino acids in 19 patients with late-life depression before and after rTMS treatment

	Before rTMS	After rTMS	*U*	*p*
Tryptophan (µmol/L)	58.6 ± 3.56	52.5 ± 2.53	0.092	n.s
Kynurenine (µmol/L)	1.85 ± 0.10	1.92 ± 0.13	0.275	n.s
Kyn/Trp (µmol/mmol)	33.3 ± 2.53	37.2 ± 2.61	0.046	n.s
Tyrosine (µmol/L)	83.9 ± 5.82	73.6 ± 5.70	0.459	n.s
Phenylalanine (µmol/L)	98.2 ± 7.22	81.4 ± 4.61	2.340	< 0.02
Phe/Tyr (µmol/µmol)	1.19 ± 0.051	1.15 ± 0.061	1.516	n.s
Nitrite (µmol/L)	45.8 ± 7.65	40.3 ± 11.4	0.872	n.s
Neopterin (nmol/L)	10.6 ± 3.43	11.7 ± 1.24	1.423	n.s

*Kyn/Trp* kynurenine to tryptophan ratio, *Phe/Tyr* phenylalanine to tyrosine ratio

Upon rTMS treatment, the phenylalanine concentrations changed significantly, they decreased to 81.4 ± 4.61 µmol/L (*U* = 2.340, *p* < 0.02), concentrations of all other biomarkers did not change significantly.

Correlations existed between serum concentrations of neopterin and Kyn/Trp (rs = 0.441, *p* < 0.02 before rTMS), results similar to earlier measurements in larger populations, indicating immune activation in late-life depression as described in patients with AD and other forms of dementia (Widner et al. 2000).

**Fig. 2:**
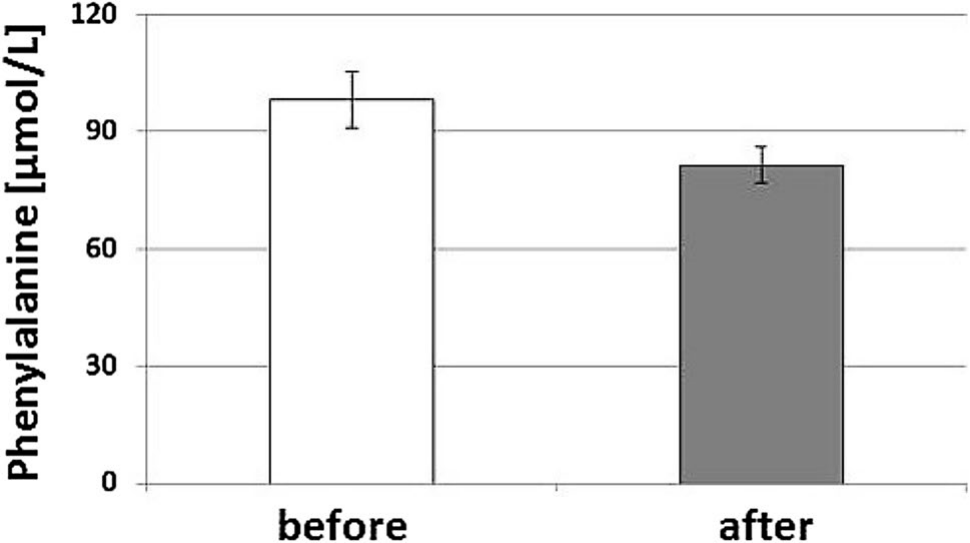
Serum phenylalanine concentrations in 19 patients with geriatric depression before and after a series of ten rTMS sessions (mean values ± SEM are shown; *U* = 2.340, *p* < 0.02)

After ten sessions of rTMS treatment, measurements of biomarkers were repeated (Table 1), and phenylalanine concentrations declined significantly (*p* < 0.02, see Fig. 2).

No significant effect was observed for any other measurement in this study (Table 1). There were also no significant effects in the SHAM group.

ANOVA with repeated measurements was designed as follows: as between subjects-variable “treatment” (i.e. verum *vs*. sham) was chosen, and as within subjects-variable, “time” (before versus after) was used. For phenylalanine, “treatment” (*F* = 8.85, 1 *df*, *p* = 0.0061) and “time” (*F* = 4.28, 1 *df*, *p* = 0.0483) were significant; the interaction term was insignificant (*F* = 0.36, 1 *df*, *p* = 0.55). For Phe/Tyr, only “treatment” (*F* = 4.55, 1 *df*, *p* = 0.422) was significant; “time” (*F* = 0.57, 1 *df*, *p* = 0.4560) and the interaction term (*F* = 0.02, 1 *df*, *p* = 0.8902) failed to reach significance. Finally, for HAM scores, “treatment” (*F* = 1.40, 1 *df*, *p* = 0.2464) was not significant in contrast to “time” (*F* = 7.52, 1 *df*, *p* = 0.0107); here, also a significant interaction term was found (*F* = 8.73, 1 *df*, *p* = 0.0064).

**Fig. 3:**
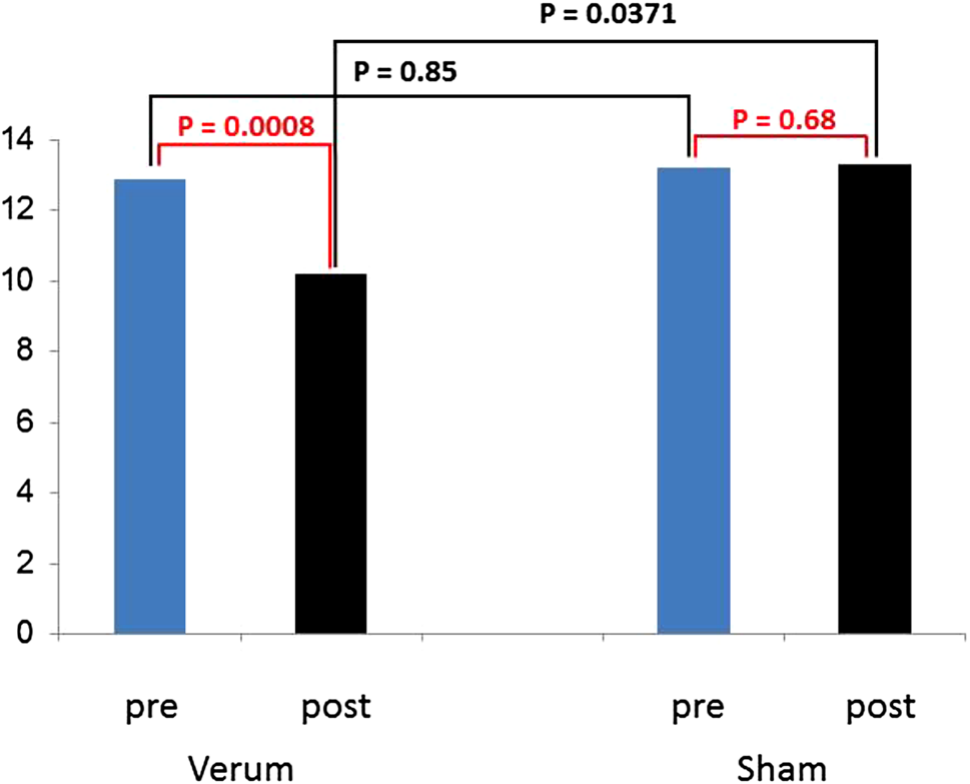
Post hoc tests: behaviour of HAM scores [all *p* values obtained by paired (red) or unpaired (black) Student’s *t* tests] in patients: the difference between pre- and post-treatment values is highly significant (*p* = 0.0008), while in controls there is practically no effect of treatment (*p* = 0.68). The difference between verum and sham pre-treatment values is not significant (*p* = 0.85); due to the significant treatment effect in patients, however, the post-treatment values differ between verum and sham (*p* = 0.0371)

Post hoc tests: Fig. 3 explains this behaviour of HAM scores [all *p* values obtained by paired (red) or unpaired (black) Student’s *t* tests]: in patients, the difference between pre- and post-treatment values is highly significant (*p* = 0.0008), while in controls there is practically no effect of treatment (*p* = 0.68). The difference between *verum* and sham pre-treatment values is not significant (*p* = 0.85); due to the significant treatment effect in patients, however, the post-treatment values differ between *verum* and sham (*p* = 0.0371).

## Discussion

rTMS is an evidence-based noninvasive treatment for depression (Shin et al. 2016; Liu et al. 2017) approved by the US Food and Drug Administration (FDA, Blumberger et al. 2018), which gave also promising results in the treatment of other neuropsychiatric disorders, such as Parkinson’s disease (von Papen et al. 2014; Dagan et al. 2017), essential tremor (Kang and Cauraugh 2017), stroke (Watanabe et al. 2018), cognitive decline and dementia (Rabey and Dobronevsky 2016; Koch et al. 2017).

Depression is a leading cause of disability worldwide contributing substantially to the global disease burden (Murray et al. 2012; Otte et al. 2016). A major challenge in treating geriatric depression is the lack of robust efficacy for many treatments that are of significant benefit to depressed working age adults (Sabesan et al. 2015).

Significant alterations of neurotransmitter levels have been previously reported in depressive syndromes (Price et al. 2009). Pro-inflammatory cytokines upregulate the biosynthesis of BH4 (Haruki et al. 2016), which is essential for the biosynthesis of serotonergic as well as adrenergic neurotransmitters a rate-limiting cofactor (Sperner-Unterweger et al. 2014).

Chronic immune activation, characteristic for depressive syndromes including geriatric depression—also seen in the herein-reported study—upregulates the production of reactive oxygen species (ROS) (Widner et al. 2000) in macrophages and destroys the oxygen labile BH4. This may lead to a reduced biosynthesis of serotonin, dopamine, adrenaline and noradrenaline, all of them important in the pathophysiology of depression (Neurauter et al. 2008; Sperner-Unterweger et al. 2014).

rTMS is an evidence-based treatment for major depressive disorder not responding to pharmacotherapy (Blumberger et al. 2018). Emerging data show positive results of rTMS in refractory geriatric depression. The evidence regarding safety as well as efficacy of rTMS in geriatric depression were discussed earlier (Galvez et al. 2015; Sabesan et al. 2015).

This exploratory intervention study further describes evidence regarding the safety and efficacy of rTMS in 19 patients with late-life depression. Patients were treated with ten consecutive sessions of rTMS within 2 weeks: rTMS induced a significant depression symptom improvement with a significant decrease in the HAMD-7 scale (*p* = 0.001) compared to 10 SHAM-treated patients. The phenylalanine concentrations simultaneously declined (*p* < 0.02) similar to earlier findings in patients with severe depression responding to electroconvulsive therapy.

Our still preliminary finding could relate to a central role of the BH4 activity in the pathophysiology of depression (Anderson et al. 1994) and may indicate that rTMS influences the enzyme PAH. PAH plays a key role in the biosynthesis of neurotransmitters noradrenaline and adrenaline which are down-stream products of tyrosine. Notably, Phe/Tyr concentrations, an index of PAH activity was not significantly influenced by rTMS. Thus, there might be another background for the decrease of phenylalanine: it could possibly relate to a change of the nutritional behaviour of patients because phenylalanine is an essential amino acid, but interestingly the concentrations of another essential amino acid, namely tryptophan, did not change significantly. Thus, it is still unclear by which mechanism rTMS contributes to the decrease of phenylalanine levels, still most probably by a functional improvement of PAH.

There are limiting factors for interpretation of this exploratory pilot study. First, only 19 patients with geriatric depression were included and treated with rTMS. Beyond that, only correlational evidence between neurotransmitter changes and noninvasive rTMS could be shown, a causative relationship still has to be verified in larger double blind SHAM controlled studies. Second, the decline of concentrations of essential amino acid phenylalanine occurred independent of any noticeable change of biomarkers of immune activation, because concentrations of neopterin and Kyn/Trp remained unchanged.

## Electronic supplementary material

Below is the link to the electronic supplementary material.

Supplementary file1 (DOC 56 kb)

Supplementary file1 (DOC 14kb)
