# Supplementary material for: Treatment of patients with geriatric depression with repetitive transcranial magnetic stimulation
Source: J Neural Transm (Vienna). 2019 Jun 27;126(8):1105–10. doi: 10.1007/s00702-019-02037-5 (PMC6647391; doi:10.1007/s00702-019-02037-5)

ANOVA with repeated measurements was designed as follows: as between subjects-variable “treatment” (i.e., verum versus sham) was chosen, and as within subjects-variable, “time” (before versus after) was used.

For phenylalanine, “treatment” ( F = 8.85, 1 df, P = 0.0061) and “time” (F = 4.28, 1 df, P = 0.0483) were significant; the interaction term was insignificant (F = 0.36, 1 df, P = 0.55).

Number of obs = 58 R-squared = 0.6185

Root MSE = 22.7853 Adj R-squared = 0.1947

Source | Partial SS df MS F Prob>F

------------+----------------------------------------------------

Model | 22729.689 30 757.65629 1.46 0.1621

|

treatment | 4842.9104 1 4842.9104 8.85 0.0061

id|treatment | 14773.732 27 547.17526

------------+----------------------------------------------------

time | 2221.4816 1 2221.4816 4.28 0.0483

time#treatment | 189.26621 1 189.26621 0.36 0.5510

|

Residual | 14017.604 27 519.17053

------------+----------------------------------------------------

Total | 36747.293 57 644.68935

For Phe/Tyr, only “treatment” (F = 4.55, 1 df, P = 0.422) was significant; “time” (F = 0.57, 1 df, P = 0.4560) and the interaction term (F = 0.02, 1 df, P = 0.8902) failed to reach significance.

Number of obs = 58 R-squared = 0.7503

Root MSE = .170164 Adj R-squared = 0.4728

Source | Partial SS df MS F Prob>F

------------+----------------------------------------------------

Model | 2.3490402 30 .07830134 2.70 0.0054

|

treatment | .33555866 1 .33555866 4.55 0.0422

id|treatment | 1.9924344 27 .07379387

------------+----------------------------------------------------

time | .01656262 1 .01656262 0.57 0.4560

time#treatment | .00056262 1 .00056262 0.02 0.8902

|

Residual | .78180291 27 .02895566

------------+----------------------------------------------------

Total | 3.1308431 57 .05492707

Finally, for HAM scores, “treatment” ( F = 1.40, 1 df, P = 0.2464) was not significant in contrast to “time” (F = 7.52, 1 df, P = 0.0107); here, also a significant interaction term was found (F = 8.73, 1 df, P = 0.0064).

Number of obs = 58 R-squared = 0.9138

Root MSE = 1.70514 Adj R-squared = 0.8181

Source | Partial SS df MS F Prob>F

------------+----------------------------------------------------

Model | 832.39392 30 27.746464 9.54 0.0000

|

treatment | 37.751815 1 37.751815 1.40 0.2464

id|treatment | 726.14474 27 26.89425

------------+----------------------------------------------------

time | 21.876679 1 21.876679 7.52 0.0107

time#treatment | 25.39392 1 25.39392 8.73 0.0064

|

Residual | 78.502632 27 2.9075049

------------+----------------------------------------------------

Total | 910.89655 57 15.980641

**Post-hoc tests and Figure**

The Figure explains this behaviour of HAM scores (all P-values obtained by paired [red] or unpaired [black] Student’s t tests): in patients, the difference between pre- and post-treatment values is highly significant (P = 0.0008), while in controls there is practically no effect of treatment (P = 0.68). The difference between verum and sham pre-treatment values is not significant (P = 0.85); due to the significant treatment effect in patients, however, the post-treatment values differ between verum and sham (P = 0.0371).
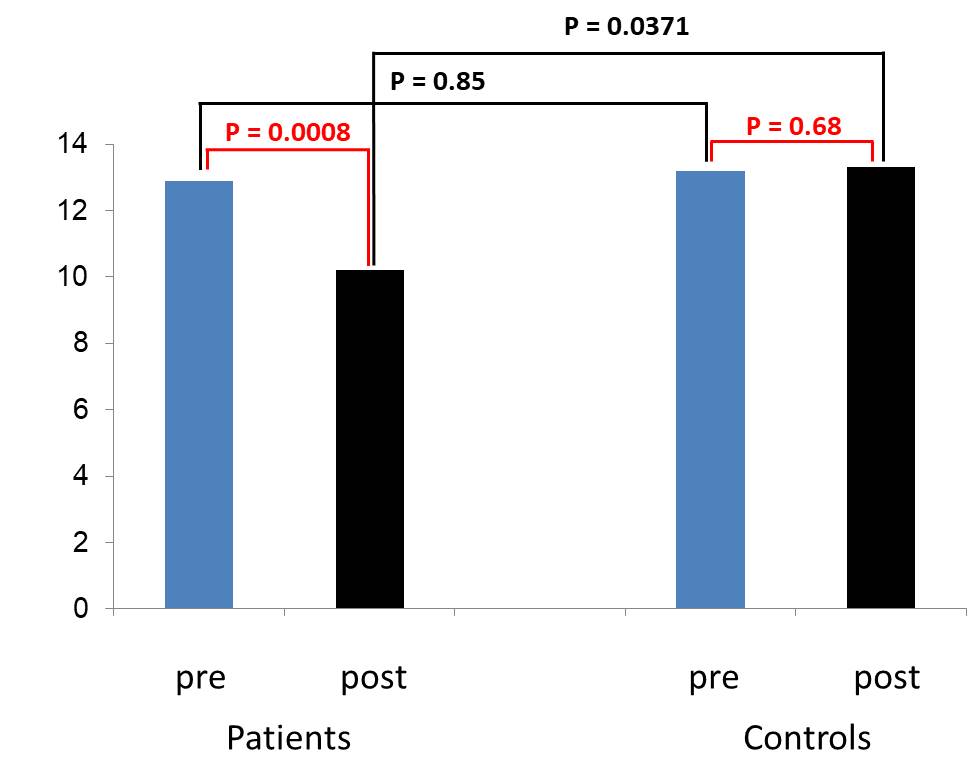

Supplement: Supplementary file 1 — Supplementary file1 (DOC 56 kb) [file 702_2019_2037_MOESM1_ESM.docx]
