# Supplementary material for: Treatment of patients with geriatric depression with repetitive transcranial magnetic stimulation
Source: J Neural Transm (Vienna). 2019 Jun 27;126(8):1105–10. doi: 10.1007/s00702-019-02037-5 (PMC6647391; doi:10.1007/s00702-019-02037-5)
Supplement: Supplementary file 2 — Supplementary file1 (DOC 14kb) [file 702_2019_2037_MOESM2_ESM.docx]

|  |  | **Phenylalanine** | **Phe/Tyr** | **HAM scores** |
| --- | --- | --- | --- | --- |
| **Model** | d.f. | 30 | 30 | 30 |
|  | F | 1.46 | 2.70 | 9.54 |
|  | Prob > F | 0.16 | 0.0054 | <0.0001 |
| **Treatment** | d.f. | 1 | 1 | 1 |
|  | F | 8.85 | 4.55 | 1.40 |
|  | Prob > F | 0.0061 | 0.0422 | 0.25 |
| **Time** | d.f. | 1 | 1 | 1 |
|  | F | 4.28 | 0.57 | 7.52 |
|  | Prob > F | 0.0483 | 0.46 | 0.0107 |
| **Time#Treatment** | d.f. | 1 | 1 | 1 |
|  | F | 0.36 | 0.02 | 8.73 |
|  | Prob > F | 0.55 | 0.89 | 0.0064 |
| **Residual** | d.f. | 27 | 27 | 27 |

d.f.: degrees of freedom; F: F statistic
